# Joint-preserving reconstruction of an acute Type IV reverse Hill–Sachs lesion following posterior shoulder fracture-dislocation: a case report

**DOI:** 10.1093/jscr/rjag995

**Published:** 2026-10-30

**Authors:** Matteo Vitali, Filippo Montalbano, Vincenzo Salini

**Affiliations:** Department of Orthopaedics and Traumatology, Ospedale San Raffaele, Via Olgettina 60, Milano MI 20132, Italy; Università Vita-Salute San Raffaele, Via Olgettina 60, Milano MI 20132, Italy; Department of Orthopaedics and Traumatology, Ospedale San Raffaele, Via Olgettina 60, Milano MI 20132, Italy; Università Vita-Salute San Raffaele, Via Olgettina 60, Milano MI 20132, Italy

**Keywords:** posterior shoulder dislocation, proximal humeral fracture-dislocation, reverse Hill–Sachs lesion, modified McLaughlin procedure, joint-preserving reconstruction

## Abstract

Posterior shoulder fracture-dislocation is a rare injury frequently associated with delayed diagnosis because of its subtle clinical and radiographic presentation. Management is primarily determined by the size of the reverse Hill–Sachs lesion, with shoulder arthroplasty generally recommended for extensive humeral head defects. We report the case of a 39-year-old man presenting with an acute posterior fracture-dislocation of the proximal humerus associated with a Type IV reverse Hill–Sachs lesion. Despite the severity of the injury, the patient underwent successful joint-preserving reconstruction using open reduction and internal fixation with cannulated screws, structural bone grafting, and a modified McLaughlin procedure. At 3-month follow-up, clinical and radiographic evaluation demonstrated a stable glenohumeral joint, satisfactory shoulder function, and painless range of motion. This case suggests that, in carefully selected young patients with reconstructable fracture fragments, anatomical reconstruction may represent a feasible alternative to primary shoulder arthroplasty while preserving native bone stock and future surgical options.

## Introduction

Posterior shoulder dislocation is an uncommon injury, accounting for ~2%–5% of all shoulder dislocations, and is most frequently associated with epileptic seizures, electrical injuries, or high-energy trauma, a combination known as the Triple E Syndrome [1]. Epileptic seizures account for nearly 50% of bilateral posterior dislocations and up to 90% of associated fractures, owing to the powerful contraction of the shoulder girdle musculature during the tonic phase [2]. Because of its subtle clinical and radiographic presentation, posterior shoulder dislocation is frequently overlooked, resulting in delayed diagnosis and treatment [3].

Patients typically present with the arm held in adduction and internal rotation, with marked limitation of external rotation [3]. Delayed diagnosis may lead to chronic pain, stiffness, instability, and degenerative arthritis [3]. Standard anteroposterior radiographs may miss the injury; therefore, axillary and scapular Y views are essential whenever posterior dislocation is suspected [3]. Computed tomography (CT) is the imaging modality of choice for defining the fracture pattern and humeral head defect, whereas magnetic resonance imaging (MRI) is useful for evaluating associated soft-tissue injuries and humeral head viability [3].

A characteristic finding is the reverse Hill–Sachs lesion, an impaction fracture of the anteromedial humeral head caused by engagement against the posterior glenoid rim [4]. Treatment depends primarily on defect size and injury chronicity [4]. Acute dislocations with defects involving ˂20%–25% of the humeral head may be successfully managed by closed reduction if stability is maintained [5]. Defects involving 20%–40% generally require joint-preserving surgery [5].

The McLaughlin procedure, consisting of transfer of the subscapularis tendon into the humeral head defect, and its modified version, transferring the lesser tuberosity together with the attached subscapularis tendon, remain established reconstructive techniques [6]. Structural bone grafting may further restore humeral head anatomy in selected young patients [6].

When humeral head involvement exceeds 40%–50%, reconstruction becomes increasingly difficult, and shoulder arthroplasty is generally recommended depending on patient age, bone quality, and glenoid involvement [6]. Chronic locked posterior fracture-dislocations have been classified into four types, with Type IV injuries representing complex comminuted fracture-dislocations that usually require prosthetic reconstruction [7]. Postoperative rehabilitation typically includes immobilization followed by progressive passive, active-assisted, and active range-of-motion exercises with subsequent rotator cuff strengthening [7].

The aim of this case report is to describe the surgical management of an acute posterior shoulder fracture-dislocation associated with an extensive Type IV reverse Hill–Sachs lesion in a young patient, in whom joint-preserving reconstruction was selected instead of primary arthroplasty. The case highlights the importance of individualized intraoperative assessment of fracture reconstructability and discusses the potential role of anatomical reduction, internal fixation, bone grafting, and the modified McLaughlin procedure in preserving the native shoulder joint in selected patients with extensive humeral head involvement.

## Case report

A 39-year-old man with a history of alcohol abuse and previous non-intravenous drug use presented to the emergency department following a fall, complaining of right shoulder swelling and pain.

Right shoulder X-ray detected displaced fracture of the right humeral head with proximal migration of the distal fragment (Fig. 1).

**Figure 1:**
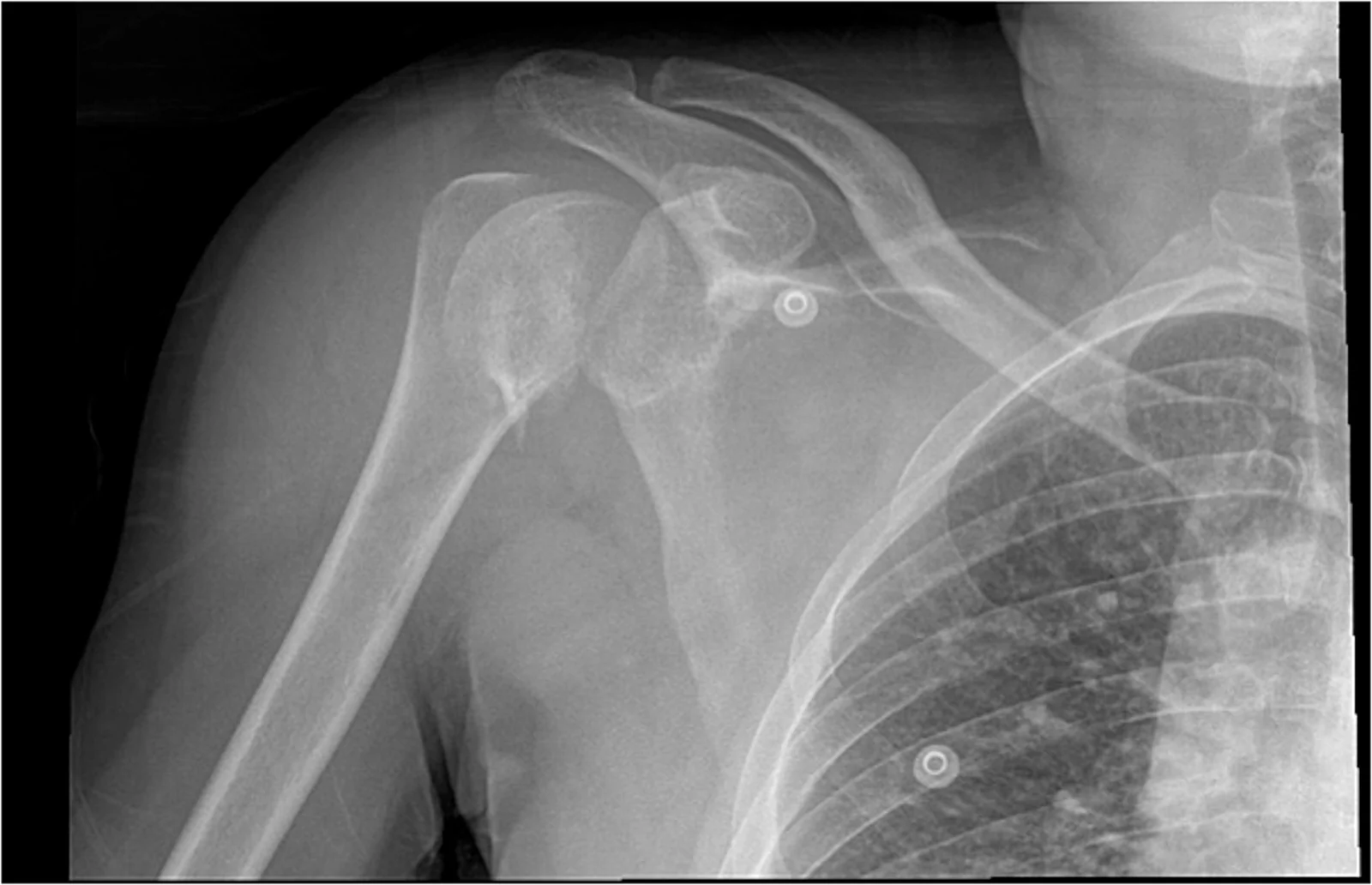
Right shoulder X-ray detected displaced fracture of the right humeral head with proximal migration of the distal fragment.

CT imaging demonstrated a comminuted displaced fracture of the proximal humerus associated with posterior dislocation of a large osseous fragment measuring ~4 cm, consistent with an acute posterior fracture-dislocation (Fig. 2). Clinical examination revealed an alert and cooperative patient with adequate pain control.

**Figure 2:**
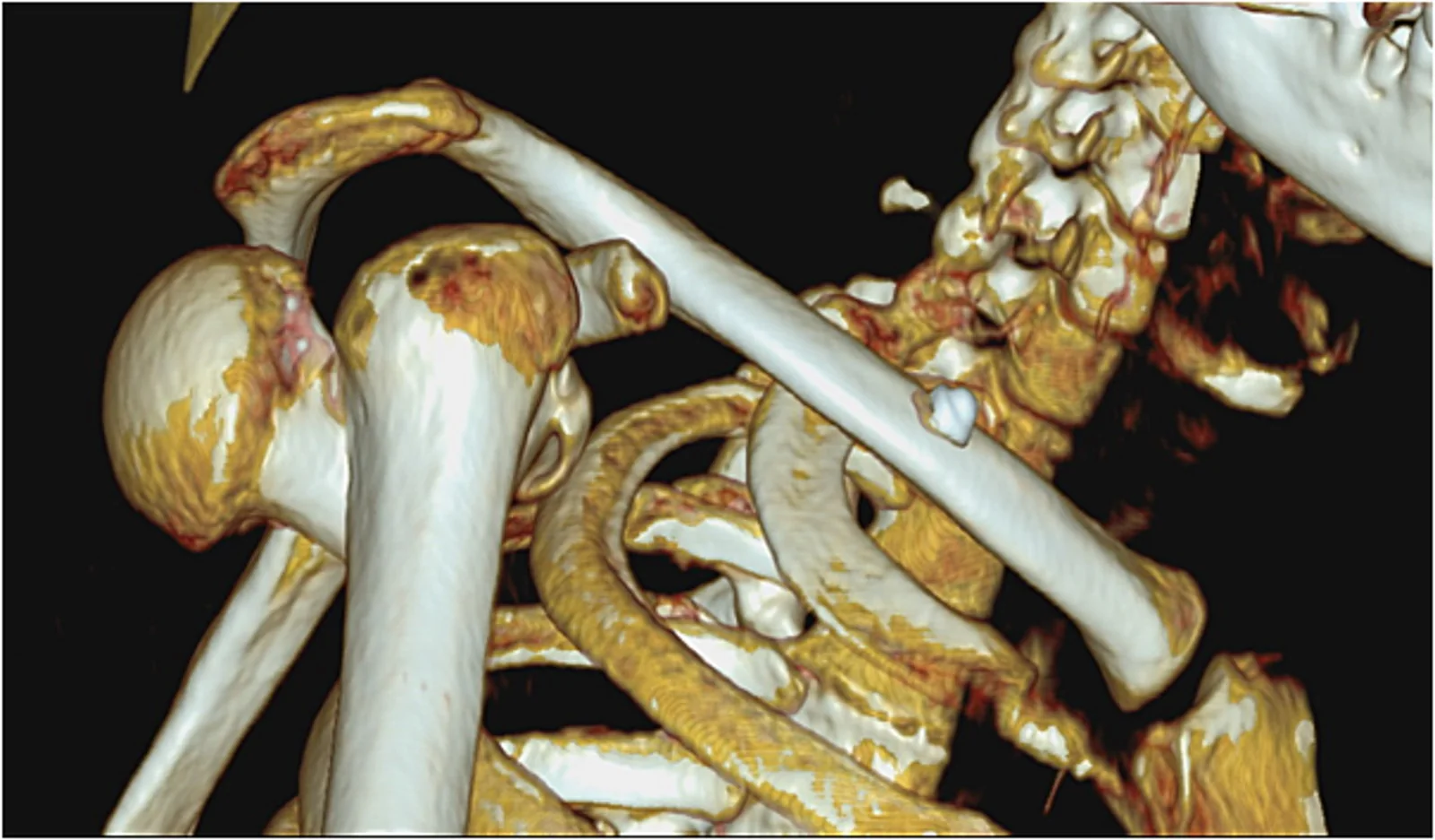
CT imaging demonstrated a comminuted displaced fracture of the proximal humerus associated with posterior dislocation of a large osseous fragment measuring ~4 cm, consistent with an acute posterior fracture-dislocation.

Considering the patient's young age and the reconstructability of the fracture fragments, a joint-preserving surgical strategy was selected despite the extensive humeral head injury.

The patient underwent surgical treatment using the modified McLaughlin technique. The procedure aimed to restore the native contour of the humeral head by impacting morselized bone allograft into the reverse Hill–Sachs defect. Through a standard deltopectoral approach, and after tenodesis of the long head of the biceps tendon, the posteriorly dislocated humeral head fragment was reduced.

Intraoperative assessment demonstrated a complex posterior fracture-dislocation with a Type IV reverse Hill–Sachs lesion (Fig. 3). Despite the extent of the humeral head injury, reconstruction was considered feasible because the articular fragments were sufficiently preserved to permit anatomical reconstruction and stable internal fixation. Fixation was achieved using four 4.0-mm partially threaded cannulated titanium screws with washers, providing stable compression and restoration of the articular contour (Fig. 4). The avulsed lesser tuberosity, together with the attached subscapularis tendon, was mobilized and transferred into the humeral head defect according to the modified McLaughlin technique and secured using two suture anchors.

**Figure 3:**
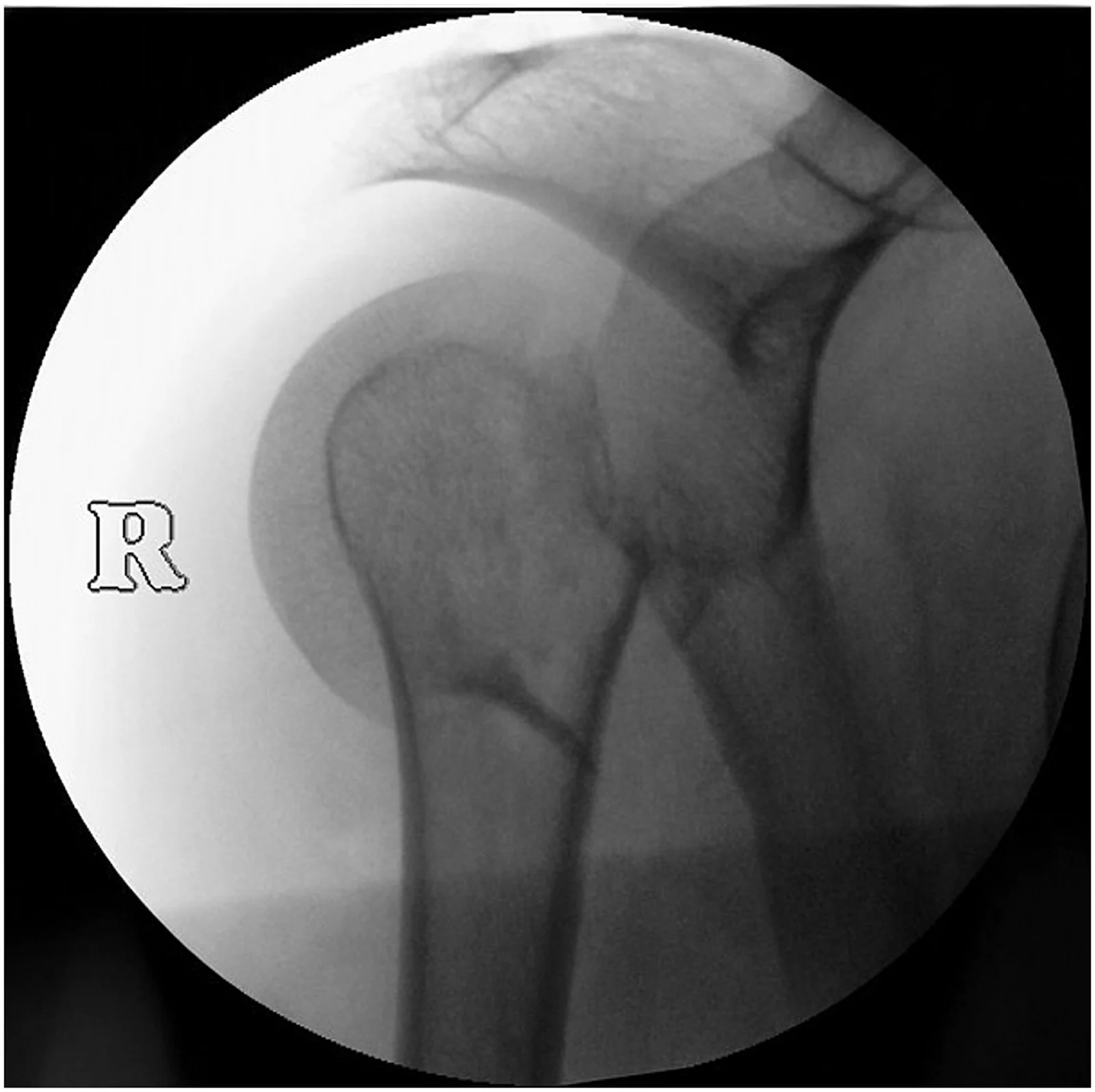
Intraoperative evaluation showing a Type IV reverse Hill–Sachs lesion.

**Figure 4:**
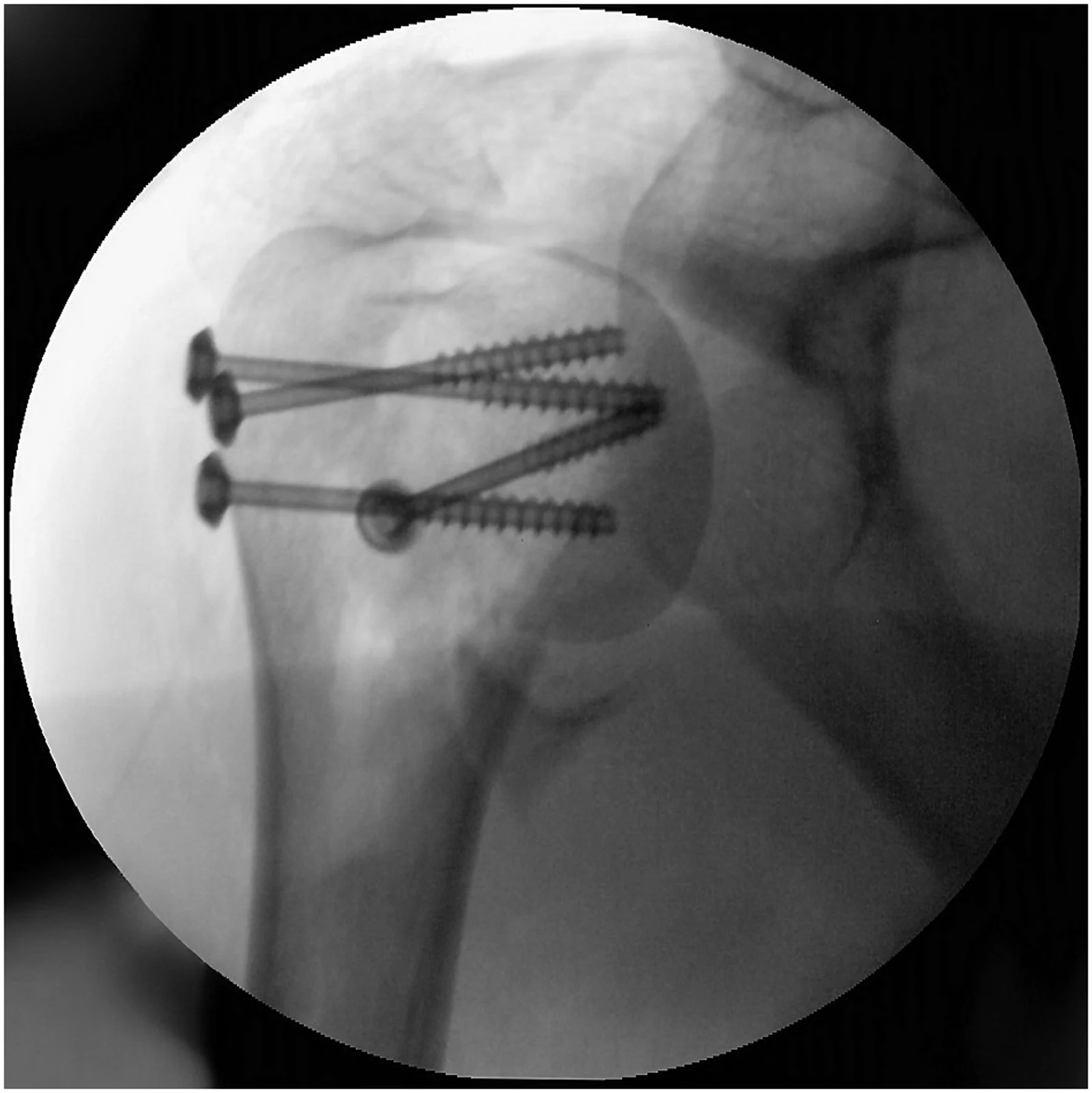
Stable fixation was obtained with four 4.0-mm partially threaded cannulated screws with washers.

For implant identification, the fixation was performed using the Asnis® III Cannulated Screw System (Stryker GmbH, Germany). Four 4.0-mm partially threaded titanium cannulated screws with corresponding washers were used, with the screws 26 mm in length. The lesser tuberosity and subscapularis tendon were secured using two 4.75-mm AlphaVent® PEEK suture anchors loaded with two strands of #2 XBraid™ S suture (REF 3910947022; Stryker Corporation, Kalamazoo, MI, USA).

Postoperative management consisted of immobilization of the shoulder in an external-rotation brace for 3 weeks, followed by a structured rehabilitation program. Passive range-of-motion exercises were initially introduced and progressively advanced to active-assisted and active movements, followed by rotator cuff strengthening over the subsequent 8 weeks. At 3-month follow-up, clinical and radiographic evaluation demonstrated a stable and congruent glenohumeral joint, painless shoulder motion, and satisfactory functional recovery.

## Discussion

This case demonstrates encouraging short-term clinical and radiographic outcomes following joint-preserving reconstruction of an acute posterior shoulder fracture-dislocation associated with a Type IV reverse Hill–Sachs lesion.

Current literature generally recommends consideration of shoulder arthroplasty for extensive humeral head defects involving ~40%–50% or more of the articular surface, particularly when the humeral head is considered irreparable. However, the extent of the defect alone should not necessarily determine the treatment strategy in every patient. In the present case, intraoperative assessment demonstrated that the articular fragments remained sufficiently preserved to permit anatomical reconstruction and stable fixation. The patient's relatively young age, the reconstructability of the humeral head, and the possibility of preserving native bone stock were therefore important factors in selecting a joint-preserving procedure rather than primary arthroplasty.

The combination of internal fixation morselized bone allograft, and the modified McLaughlin procedure allowed restoration of the humeral head contour while maintaining the native glenohumeral joint. This approach should not be interpreted as a general alternative to arthroplasty for all Type IV injuries, but rather as a potential option in carefully selected patients in whom anatomical reconstruction is technically achievable. Previous reports have similarly described the modified McLaughlin procedure as a joint-preserving strategy for posterior fracture-dislocations with substantial reverse Hill–Sachs defects, particularly when the lesser tuberosity and subscapularis can be incorporated into the reconstruction. The principal advantage of this approach lies in preservation of the native humeral head, maintaining bone stock and avoiding the immediate disadvantages of prosthetic replacement in a young patient. In addition, should reconstruction fail or secondary degenerative changes develop, shoulder arthroplasty remains available as a salvage procedure without compromising future treatment options.

The patient's history of alcohol abuse and previous recreational drug use was considered as part of the overall clinical and social assessment. He denied intravenous drug use, and these factors did not constitute, in themselves, an indication for prosthetic replacement. The decision to pursue joint-preserving reconstruction was primarily based on the patient's age, the acute nature of the injury, the preservation and reconstructability of the articular fragments, and the possibility of achieving stable anatomical fixation.

The patient's social history was relevant to the postoperative management of a complex reconstructive procedure. Alcohol and previous substance use may potentially influence adherence to immobilization, activity restrictions, rehabilitation, and scheduled clinical follow-up. In the present case no specific modification of the rehabilitation protocol was required. The patient completed the early postoperative rehabilitation period with satisfactory clinical and radiographic findings at three months.

These observations should be interpreted cautiously because the present report describes a single patient and the short follow-up period does not allow conclusions regarding the long-term influence of the patient's social history on adherence, rehabilitation, or complications. Nevertheless, documenting these factors is relevant because successful joint-preserving reconstruction depends not only on the technical feasibility of restoring the humeral head but also on adequate postoperative protection and rehabilitation.

The present report has several limitations that should be acknowledged. First, it describes a single patient, limiting the generalizability of the findings. Second, the follow-up period was limited to three months and therefore does not permit assessment of longer-term complications, including avascular necrosis, collapse of the reconstructed humeral head, post-traumatic osteoarthritis, implant-related complications, or the eventual need for salvage arthroplasty. Third, the absence of a comparative group prevents direct comparison with primary arthroplasty or other reconstructive techniques. Finally, although the patient's history of alcohol abuse and previous drug use was documented and considered during postoperative management, a single case cannot establish whether these social factors influence compliance, rehabilitation, or clinical outcomes after complex joint-preserving shoulder reconstruction.

## Conclusion

Posterior shoulder fracture-dislocation remains a rare but clinically significant injury requiring a high index of suspicion and appropriate imaging for timely diagnosis. Although shoulder arthroplasty remains the standard treatment for extensive humeral head defects, this case demonstrates that joint-preserving reconstruction combining anatomical reduction, internal fixation, structural bone grafting, and the modified McLaughlin procedure can provide satisfactory short-term outcomes in carefully selected young patients with reconstructable Type IV reverse Hill–Sachs lesions. Further studies with larger cohorts and longer follow-up are required to define the indications and long-term effectiveness of this bone-preserving strategy.
